# Inhibitory Control Development: A Network Neuroscience Perspective

**DOI:** 10.3389/fpsyg.2022.651547

**Published:** 2022-10-10

**Authors:** Weixi Kang, Sònia Pineda Hernández, Md. Shahinoor Rahman, Katharina Voigt, Antonio Malvaso

**Affiliations:** ^1^Computational, Cognitive and Clinical Neuroimaging Laboratory, Division of Brain Sciences, Department of Medicine, Imperial College London, London, United Kingdom; ^2^Euncet Business School, Polytechnic University of Catalonia, Barcelona, Spain; ^3^Department of Psychology, University of Chittagong, Chittagong, Bangladesh; ^4^School of Psychological Sciences and Turner Institute for Brain and Mental Health, Monash University, Melbourne, VIC, Australia; ^5^Monash Biomedical Imaging, Monash University, Melbourne, VIC, Australia; ^6^School of Medicine and Surgery, Vita-Salute San Raffaele University, Milan, Italy; ^7^Neuroimaging Research Unit, Division of Neuroscience, Scientific Institute for Research, Hospitalization and Healthcare (IRCCS) San Raffaele Scientific Institute, Milan, Italy; ^8^Neurology Unit, Scientific Institute for Research, Hospitalization and Healthcare (IRCCS) San Raffaele Scientific Institute, Milan, Italy

**Keywords:** executive function, inhibitory control in children, inhibitory control in adolescents, rIFG, inhibition, inhibitory control development

## Abstract

As one of the core executive functions, inhibition plays an important role in human life through development. Inhibitory control is defined as the ability to suppress actions when they are unlikely to accomplish valuable results. Contemporary neuroscience has investigated the underlying neural mechanisms of inhibitory control. The controversy started to arise, which resulted in two schools of thought: a modulatory and a network account of inhibitory control. In this systematic review, we survey developmental mechanisms in inhibitory control as well as neurodevelopmental diseases related to inhibitory dysfunctions. This evidence stands against the modulatory perspective of inhibitory control: the development of inhibitory control does not depend on a dedicated region such as the right inferior frontal gyrus (rIFG) but relies on a more broadly distributed network.

## Introduction

Inhibitory control refers to the ability to suppress prepotent actions when they are unlikely to accomplish valuable results (Bari and Robbins, 2013) and is a core executive cognitive function (Diamond, 2013). Intact inhibitory control abilities ensure behaviors are consistent with one’s intentions and motivations but suppress irrelevant or inappropriate responses (Miller and Cohen, 2001). A loss of inhibitory control tends to cause diseases characterized by poor impulse control, even though the equation loss of inhibition equals to inability to control urge is faulty. Inhibitory control does not refer to a single executive function but consists of several components (Tiego et al., 2018; Mirabella, 2021). Distinctions between motor and interference inhibition are widely recognized. The ability to inhibit a preplanned motor response is known as motor inhibition, and it is typically assessed using the go/no-go or stop-signal (SST) tasks, both of which require participants to respond to target (“go”) stimuli using a motor response while inhibiting responses to relevant (“no-go”) stimuli (Congdon et al., 2012). Interference inhibition, on the other hand, measures the capacity to overcome reaction conflict caused by irrelevant but incompatible stimulus attributes that must be inhibited to prevent incorrect responses. The Stroop (1935), Simon (Simon and Rudell, 1967), Flanker (Eriksen and Eriksen, 1974), and Antisaccade (AS; Munoz and Everling, 2004) tasks are commonly used to investigate this form of inhibitory control. There are at least two neuropsychological areas of motor inhibition: (1) reactive inhibition, or the ability to halt a reaction automatically when a stop instruction is offered; and (2) proactive inhibition, or the ability to adapt a motor approach to the context in which a person is embedded. In disorders characterized by a lack of impulse control, both components are likely to play a role (Mirabella, 2021). One of the major goals of contemporary cognitive neuroscience is to understand the neural basis underlying distinct cognitive processes. In the context of inhibitory control, there are primarily two schools of thoughts: a modular perspective and a network account (Aron et al., 2014; e.g., see Hampshire and Sharp, 2015 for a review). Specifically, the modular perspective proposes that the right inferior frontal gyrus (rIFG) is a dedicated region for behavioral inhibition (e.g., Aron et al., 2003). By contrast, a network account proposes that just like other executive functions, inhibitory control is supported by domain-general brain regions, such as the frontal multiple-demand (MD) cortex, which activates at a variety of cognitive tasks and includes the rIFG (Duncan, 2010).

Impaired inhibition control is a central facet of many psychiatric disorders, including attention-deficit hyperactivity disorder (ADHD; Pliszka et al., 2000; Malloy-Diniz et al., 2007), alcohol and substance-use disorders (Verdejo-García et al., 2008; Dick et al., 2010), borderline personality disorder (Barker et al., 2015), and neurological disorders, such as Parkinson’s Disease (PD; Evans et al., 2009). Moreover, impaired inhibition control predicts poorer therapy result and retention (Loree et al., 2015), as well as poorer everyday functioning among clinical populations (Ellis et al., 2004; Tomko et al., 2014). Impaired inhibition is related with risk-taking behaviors among clinical and non-clinical populations, including reckless driving/driving under the influence (Bıçaksız and Özkan, 2016; Luk et al., 2017) and risky sexual behaviors (Dir et al., 2014). Given the various negative consequences of poor inhibition control, there has been a significant effort to define and comprehend inhibition control as a construct. However, how it evolves across human development has been understudied.

Yet, understanding the developmental trends of inhibitory control is critical when aiming to reduce the adverse outcomes and burdens related to inhibition control. Further, fostering the development of inhibition in children can have positive outcomes. For example, inhibitory control plays a central role in predicting social-emotional competence. Children who had better inhibitory control abilities were more likely to have better social skills and less internalizing behaviors (Liu et al., 2018). Moreover, inhibitory control abilities appear to be related to both math and literacy skills in young children. The strength of the association between inhibitory control skills and academic performance is similar between preschoolers and kindergarteners (Allan et al., 2014).

The aim of this systematic review is to provide a qualitative summary of existing behavioral and neuroscientific investigation of inhibitory control during development. This systematic review is structured as follows: after detailing methods of conducting this systematic research and outlining the results, we list and discuss the paradigms used in inhibitory control research and review behavioral results from research focused on inhibitory control development. Next, we survey the neural basis of inhibitory control both from a modulatory and a network perspective. Then, we discuss the neuroimaging literature regarding the brain activation patterns from infants to teenagers and argue that inhibitory control is supported by domain-general regions just like other cognitive functions. After that, we considered what if inhibitory control ability goes wrong by characterizing various neurodevelopment diseases associated with inhibitory control. Finally, we conclude our study and provide directions for future research.

### Paradigms Used in Inhibitory Control Research

In the literature, the Simon task (Simon and Rudell, 1967), the Stroop task (MacLeod, 1991), the AS (Munoz and Everling, 2004), the go/no-go task (Cragg and Nation, 2008), the Flanker task (Eriksen and Eriksen, 1974; Mullane et al., 2009), the SST (Verbruggen and Logan, 2008), and the masked priming task (Keute et al., 2018) are typically used to measure inhibitory control development. Among them, the Simon task, the Stroop task, the AS, and the Flanker task measure interference inhibition whereas the go/no-go task, the SST, and the masked prime task are used to measure motor inhibition (Mirabella, 2021). Individuals must complete the Stroop task by looking at a list of words that are printed in a distinct or the same color rather than based on the meaning of each word. There are two kinds of trials in the Stroop task: congruent and incongruent. In the congruent trials, the presented color words (e.g., “red”) are exactly the same as the color of the ink (e.g., red). By contrast, in the incongruent trials, the color words (e.g., “red”) are different from the color of the ink (e.g., green). Participants were required to report the ink color, but to ignore the meaning of the word. Findings showed that participants are slower and make more errors in the incongruent trials when they are requested to report the color of the ink, not when they have to report the meaning of the words.

Simon tasks have two very simple rules: (1) press the left button when seeing Stimulus A and (2) press the right when seeing Stimulus B. The stimuli appear one at a time, and they can be presented on either the right or left side of the screen. Participants tend to be slower when the stimulus is given on the side opposite the corresponding response. This phenomenon is also known as the Simon effect, which states that people have a strong inclination to respond on the same side as the stimulus (Lu and Proctor, 1995; Hommel, 2011). As a result, in incompatible trials, the irrelevant dimension activates an incorrect response propensity, which must be resisted because it interferes with the selection of the proper response.

The Flanker task asks participants to attend to the stimulus presented in the center but to ignore the flanking stimuli close to it. In incongruent trials, where the surrounding stimuli are mapped to the opposite reaction as the stimulus in the center, people respond slower; thus, inhibitory control ability is needed to overcome the tendency to respond according to the flanking stimuli. All these abovementioned tasks measure the so-called interference inhibition.

By contrast, the go/no-go task, the SST, and the masked prime task are used to measure motor inhibition (Mirabella, 2021). The go/no-go task and the SST are two of the most prevalent behavioral paradigms in neuroimaging research for measuring response inhibition. The primary task in both paradigms is either a basic or a choice reaction task. Depending on whether a go or no-go stimulus is supplied, participants must respond (by hitting a specified key) or withhold a response (by not pushing a designated key) in the go/no-go task. The SST instructs participants to respond to a go-signal as quickly as possible, but to inhibit this planned movement in response to an infrequent stop-signal (typically a sound or a visual stimulus) supplied at various delays following the go signal. These two tasks differ due to the fact the former assesses the ability to prevent an action from occurring (i.e., action restraint) the latter assesses the ability to prevent an action that has already been initiated from occurring (i.e., action cancelation). All trials in the masked priming task start with the presentation of a fixed dot (Keute et al., 2018). Then, for the shortest duration feasible (one refresh rate of the display), participants are shown an arrow, followed by a longer-lasting random pattern mask (i.e., a random mix of vertical and horizontal lines), rendering the preceding stimulus (the prime) unconsciously. Finally, a supraliminal arrow target is present. Participants must press the right key if the supraliminal arrow goal points to the right, and the left key if it points to the left. When the prime arrow points in the same direction as the arrow target, the trial is considered compatible; otherwise, it is considered incompatible (Keute et al., 2018).

## Methods

### Systematic Review

#### Search Strategy

A literature search was conducted on the PubMed database^1^ on 14 September 2021 using the following Boolean search string: *(Inhibitory control OR inhibition) AND (Development OR Infant OR Children OR Adolescent) AND (Neuro OR Neural).* A publication year limit has been set from 2000 to 2021. All returned results were systematically identified, screened then extracted for relevant information following the PRISMA *(Preferred Reporting Items for Systematic Reviews and Meta Analysis)* guidelines.^2^

#### Inclusion and Exclusion Criteria

Studies that fulfilled the following criteria were included in our systematic review: (a) inhibitory control behavioral performance during development, (b) neural basis of inhibitory control during development, (c) neurodevelopmental disease related to inhibitory dysfunction.

Studies falling into one or more of the following categories were excluded from further analysis: (a) not original research article (e.g., review, opinion article, or conference abstract), (b) used non-human organisms (e.g., primates, rodents), and (c) not related to inhibitory control development mechanisms or inhibitory control behavioral performance during development.

## Results

**FIGURE 1:**
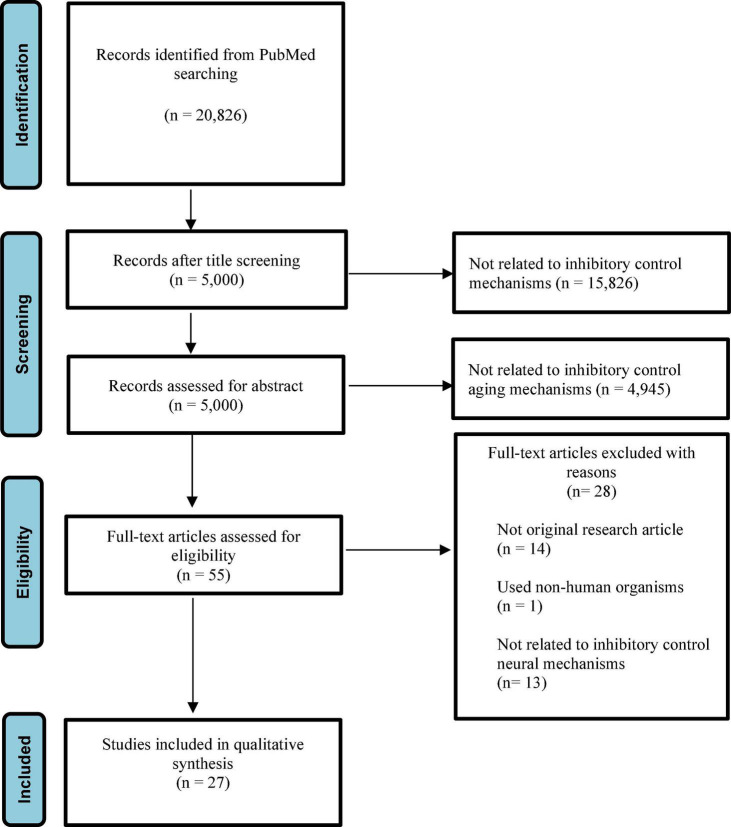
The PRISMA chart that shows the process followed to select the included studies.

Among the 20,826 articles initially turned after search on PubMed, we selected 5,000 articles after initial title screening. In particular, we excluded 15,825 articles because they did not relate to inhibitory control mechanisms. Then, records left for abstract screening were 5,000. After screening, we excluded 4,945 studies because they did not relate to inhibitory control aging mechanisms, with 55 left with eligibility. We assessed the full text of these articles and excluded 25 articles because 1 of them did not use human participants, 14 of them were not original research articles, and 13 of them did not relate to inhibitory control neural mechanisms. Therefore, 27 articles were left for our systematic review (Figure 1). Results were summarized in Table 1.

**TABLE 1:** Information about studies, development stage, N, methods, tasks employed, and brain regions recruited/activated.

Study	Development stage	N	Methods	Tasks	Brain regions recruited/activated
Bell (2001)	Infants	54	EEG	Visuo-spatial task	Right frontal parietal cortex (FPC)

Morasch and Bell (2011)	Toddlers	81	EEG	Conflict task, inhibitory control delay task, and inhibitory control compliance task	Left and right lateral frontal scalp (LFS)
Watson and Bell (2013)		68	EEG	Nonverbal inhibitory control task, day-night Stroop-like task, less is more task, and hand game task	Right and left medial frontal cortex (MFC)

Pliszka et al. (2000)	Children	20	EEG	SST	Right inferior frontal cortex (IFC)
Bunge et al. (2002)		16	fMRI	Go/no-go task	Bilateral precuneus (BLPcu), left angular gyrus (AnG), right middle temporal gyrus (mTG), right middle frontal gyrus (mFG), and left ventrolateral prefrontal cortex (VLPPFC)
Durston et al. (2002)		10	fMRI	Go/no-go task	Ventral-striatal regions (VStR), bilateral ventral prefrontal cortex (BLVPFC), right parietal lobe (PL), and right dorsolateral prefrotal cortex (dlPFC)
Wolfe and Bell (2004)		75	EEG	Day/night Stroop-like task and the yes/no task	Prefrontal cortex (PFC) and left-right medial frontal cortex (MFC)
Jonkman et al. (2007)		33	EEG	Go/no-go task	Right medial frontal cortex (MFC)
Schel et al. (2014)		19	fMRI	Marble task	Right fronto-basal ganglia network (FBGN), subthalamic nucleus (STN), and right dorsal fronto-median cortex (DFMC).
Rahman et al. (2017)		31	EEG	Go/no-go task	Left frontal N2 and posterior P3.

Tamm et al. (2002)	Teenagers	19	fMRI	Go/no-go task	Left inferior frontal gyrus (IFG), insula and orbitofrontal gyrus
Durston et al. (2006)		14	fMRI	Go/no-go task	Bilateral ventral prefrontal cortex (BLVPFC), right inferior frontal gyrus (IFG), bilateral anterior cingulate gyrus (BLACG), right superior frontal gyrus (SFG), right inferior prefrontal cortex (IPFC), and right precentral gyrus (PrcG)
Smith et al. (2006)		46	fMRI	Go/no-go task, Stroop task and switch task	Left rostral mesial frontal cortex (RMeFC), bilateral prefrontal cortex (BLPFC), temporal lobe (TL), and right parietal lobe (PL)
Rubia et al. (2007)		26	fMRI	SST	Right inferior prefrontal cortex (IPFC), thalamus, striatum, cerebellum, and anterior cingulate gyrus (ACG)
Carrion et al. (2008)		30	fMRI	Go/no-go task	Left middle frontal cortex (mFC) left cuneus, left inferior occipital gyrus (IOG), left inferior temporal gyrus (ITG), right and left medial frontal gyrus (MFG), right and left anterior cingulate gyrus (ACG)
Vidal et al. (2012)		14	MEG	Go/no-go task	Left middle frontal gyrus (mFG), left inferior frontal gyrus (IFG), right prefrontal cortex (PFC), and right middle temporal gyrus (mTG)
Bhaijiwala et al. (2014)		24	fMRI	SST	Right medial prefrontal cortex (MPFC) and right inferior frontal gyrus (IFG)
Triplett et al. (2014)		34	fMRI	Antisaccade task	Bilateral frontal eye field (BLFEF), supplementary eye fields (SUEF), putamen, and precuneus, and left posterior cingulate gyrus (PtCG)
Vara et al. (2014)		15	MEG MRI	Go/no-go task	Left inferior frontal gyrus (IFG) right middle temporal gyrus (mTG), right superior temporal gyrus (STG) right precentral gyrus (PreG), and right parietal lobule (PL)
Padmanabhan et al. (2015)		20	fMRI	AS	Left putamen, left precuneus, right inferior parietal lobule (IPL)
Marek et al. (2015)		135	fMRI	AS	Cingulo-opercular
Kim-Spoon et al. (2016)		157	fMRI	Multiple Source Interference Task	Left posterior medial frontal cortex (PtMFC)
Hwang et al. (2016)		17	MEG	AS/prosaccade task	Right dorsolateral prefrontal cortex (dlPFC) frontal eye field (FEF)
Voorhies et al. (2018)		224	fMRI	Go/no go task	Right inferior frontal junction (IFJ), posterior cingulate gyrus (PtCG)
Bartholdy et al. (2019)		172	fMRI	SST	Medial prefrontal cortex (MPFC) and anterior cingulate cortex (ACC)
Quach et al. (2020)		113	fMRI	AS	Left posterior parietal cortex (PtPC), left middle frontal gyrus (mFG), and left inferior parietal lobule (IPL), left angular gyrus (AnG)

Petrican and Grady (2019)	22-36 years old and 12-85 years old	359 and 247	fMRI	N-back task, social cognition task, and incentive processing task	Default Mode Network, frontoparietal network and salience network

### Behavioral Evidence Suggesting the Development of Inhibitory Control

Previous studies showed that inhibitory control develops across the lifespan. Inhibitory control develops throughout childhood (Van de Laar et al., 2014; Cragg, 2016). It has been proven that inhibitory control ability reaches complete maturation around the age of 12 or later (Tipper et al., 1989; Diamond et al., 1994; Gerstadt et al., 1994; Enns et al., 1998; Van der Meere and Stemerdink, 1999; Rubia et al., 2000; Carver et al., 2001; Bunge et al., 2002). Compared to childhood, further improvements in inhibitory control during adolescence are only subtle (Bedard et al., 2002; Luna et al., 2004; Huizinga et al., 2006; Ordaz et al., 2013).

According to the other studies, nine-month-old infants may pick between concurrently presented places by suppressing reaction to the distractor site, similarly to adults (Johnson, 1995; Amso and Johnson, 2005). Inhibitory control abilities increase dramatically throughout childhood, as evidenced by a number of inhibitory control tasks. The Flanker task performance improved significantly during childhood, but just somewhat into puberty (Cragg, 2016). Likewise, the capacity to halt develops dramatically during childhood, reaching practically adult-like performance by adolescence, as tested by the SST (Huizinga et al., 2006), the go/no-go task (Lewis et al., 2017), and the Stroop task (Adleman et al., 2002). These findings, taken together, provide persuasive evidence that the ability to use executive systems to inhibit responses is acquired gradually during development. In line with these, Williams et al. (1999) used the SST to investigate the development of inhibitory control ability. There were 275 participants ranging from 6 to 81 years of age participating in the study. Williams et al. (1999) found that the speed of processing of stopping increased with age through childhood. Notably, some evidence shows that reactive, but not proactive inhibitory control, decreases with aging (e.g., Smittenaar et al., 2015).

Wiebe et al. (2012) studied the development of reaction inhibition in preschoolers and found factors that related to individual differences in response inhibition changes across time. The go/no-go assignment was completed by the children at various times throughout the preschool term. There were significant improvements in both accuracy and speed from 3 to 5.25 years, but the trajectories were extremely diverse and revealed distinct patterns of connections with predictors. No-go trials were more challenging for children than go trials, indicating that the attempt to develop a prepotent tendency to respond was successful. The growth of accuracy which reflects children’s differential responses to go and no-go stimuli, was mostly linear, with a small non-linear component reflecting decelerating accuracy later in the preschool years; relationships with predictors were simple, with better working memory and IQ predicting more accurate responses. The reaction speed trajectory, on the other hand, was predominantly curved, suggesting a slowdown of children’s answers between the ages of 3 and 3.75, followed by faster replies in the remaining assessments. Male sex and higher surgency predicted faster responses throughout the preschool period, although superior working memory and IQ predicted slower responses at younger ages, but faster responses in older preschool children.

### Modular vs. Network Perspectives in Understanding Inhibitory Control

Understanding the functional brain architectures underlying human cognition is one of the major goals of modern cognitive neuroscience, which can be done by localizing different cognitive processes to distinct brain regions and their connection pathways. For example, if Brain Region A activates in Task 1, then the brain region should be considered to support Task 1 according to the localists’ view. In the context of inhibitory control, a predominant theory proposed that the rIFG/anterior insula (aIns) is dedicated to inhibitory control (Aron et al., 2003). Evidence that supports this inhibitory control hypothesis came from (1) neuroimaging studies that have reported increased activations in the rIFG/aIns when participants canceled their stop response in the SST (Rubia et al., 2001). (2) Clinical studies that found patients were unable to inhibit when these brain regions had abnormal activities. Moreover, lesions in the rIFG/aIns were associated with disinhibition (Aron et al., 2003).

In a later review, Aron et al. (2014) revised the modular inhibitory control hypothesis by suggesting that the rIFG/aIns and their interactions with the pre-supplementary motor areas are important for inhibition (Aron et al., 2014). These two regions would impose inhibition via projections to the right subthalamic nucleus (STN), which would subsequently decrease activities in the premotor cortex and primary motor cortex (Coxon et al., 2006; Mirabella et al., 2011; Mattia et al., 2012, 2013). In any case, the idea of a right-lateralized network (particularly the rIFG) serving as an inhibitory control mechanism has been seriously questioned (see Mirabella, 2014 and Hampshire and Sharp, 2015 for reviews). First of all, the left inferior frontal gyrus has been revealed to play a significant role in inhibitory control (Swick et al., 2008). Second, deep brain stimulation (DBS) of the subthalamic nucleus has been found to restore reactive inhibitory control to a near-normal level in both bilateral (Mirabella et al., 2012, 2013) and unilateral (Mancini et al., 2019) cases. Third, Mirabella et al. (2017) compared the inhibitory efficiency of right and left dominance of symptoms in Parkinson’s disease (RPD and LPD, respectively) in the middle stages of the disease (Hoehn and Yahr -2 or -3), but found no differences in reactive or proactive inhibition between LPD and RPD patients, despite the fact that patients were affected compared to healthy controls. This conclusion was recently verified by testing PD patients in the early stages of the disease, when the condition is unilateral (Di Caprio et al., 2020). Overall, the evidence suggests that inhibitory control is not solely a function of the right hemisphere, but rather of collaboration between the two.

Network perspective proposed that the inhibitory control is one of the examples of cognitive control, which relies on the same set of domain-general fronto-parietal networks (see Hampshire and Sharp (2015) for a review). The network perspective argues that the attempt to map the inhibitory control ability to a dedicated brain region is misleading because both the SST and go/no-go task do not control for potential confounding cognitive demands (Mostofsky et al., 2003; Shallice et al., 2008; Hampshire et al., 2010; Munakata et al., 2011; Walther et al., 2011; Criaud and Boulinguez, 2013). Therefore, the evidence that supports the modular inhibitory control hypothesis equates to a proof that brain representation in inhibitory control has not been established. Indeed, literature has reported the rIFG is involved in a wide variety of tasks that are attention demanding but has no requirement for inhibitory control (Hampshire et al., 2010, 2011, 2013; Munakata et al., 2011). Florence et al. (2014) included several control conditions where no inhibitory demands were needed and compared them to the brain activities when participants were doing the SST tasks. Their findings showed that the appropriate IFC subregions not only activated when inhibitory cognitive demands were present, but also constituted components of spatially distributed networks. Furthermore, even though behavioral inhibition was not required, these networks were substantially active when individuals were processing infrequent inputs and learning new tasks (Florence et al., 2014). Mirabella (2014), for example, hypothesized that acting and halting are functions emerging from complex interactions across overlapping brain regions, the behavior of which is linked to evaluations of the benefits and drawbacks of an action. Thus, the inhibitory network (along with the one that serves the other executive functions) is not restricted to the “frontal multiple-demand cortex” but extends to a number of cortical and subcortical brain areas. As a result, the modular inhibitory control hypothesis should be dismissed, while the network perspective should be preferred.

### Changes of Inhibitory Control During Development

#### Infants (Under 2 Years Old)

Studies have shown that the electroencephalogram (EEG) activities recorded from frontal skeletal regions affect newborns’ inhibitory control skills (Bell and Morasch, 2007). Furthermore, task-related EEG activity variations from a baseline state would reflect changes in cortical functioning related to task performance (Pivik et al., 1993). Infants who did well on a visuo-spatial inhibitory control task had task-related alterations in EEG activity in the frontal-parietal region of the right hemisphere. Infants with less developed inhibitory control abilities, on the other hand, showed no differences in EEG activity from baseline to task (Bell, 2001).

#### Toddlers (2–4 Years Old)

One study examined the inhibitory control abilities in 81 toddlers aged from 24 to 27 months (Morasch and Bell, 2011). Specifically, Morasch and Bell (2011) applied maternal report measure and a battery of inhibitory tasks, including the conflict task, the inhibitory control delay task, the inhibitory control compliance task to assess the inhibitory control abilities of toddlers in the EEG session. Morasch and Bell (2011) found that the variances in the inhibitory control abilities can be explained by the electrical signals at the left and right lateral frontal scalp site and the laboratory inhibitory task performance. Another study by the same group subsequentially linked inhibitory control to medial frontal electroencephalographic activity in both hemispheres (Watson and Bell, 2013).

#### Children (4–12 Years Old)

Similar findings were obtained in studies evaluating the biobehavioral expression of inhibitory control in two groups of participants aged 4 and 4.5 years old. Children who performed better on inhibitory control tasks (such as the day/night task and the yes/no Stroop task) had changes in EEG activities in the medial frontal cortex, indicating prefrontal cortex activation in both hemispheres. Preschoolers who performed poorly on the task, on the other hand, showed no task-related increases in EEG activity (Wolfe and Bell, 2004, 2007).

Bunge et al. (2002) examined the inhibitory control ability in children aged 8–12 years old with go/no-go tasks. They found that the performance depends on the recruitment of a subset of the network that was recruited during adult response inhibition. More specifically, effective performances in children recruited posterior regions in both hemispheres, but not frontal regions as activated in adults. This suggested that the functional network of adults for response inhibition is partially recruited in children, and this develops with age (Bunge et al., 2002). Bunge et al. (2002) also included a condition where they could study cognitive control ability (interference suppression) in these participants.

Bunge et al. (2002) found that increases in inhibitory control capacity in children aged 8–12 were linked to activations in a subset of posterior brain areas that were consistently active in adulthood. Brain activities in the bilateral precuneus, left angular gyrus, right middle temporal gyrus, and right middle frontal gyrus were linked to children’s performance. Furthermore, children’s activities in posterior association areas were a better predictor of performance than prefrontal regions. In other words, poor performance was linked to activation of the left ventrolateral and bilateral dorsolateral prefrontal cortex (DLPFC), whereas good performance was linked to bilateral inferior parietal activation. It’s possible that the prefrontal actions in children who performed poorly were related to the tactics they utilized, rather than being important to their capacity to suppress during the task. Moreover, they discovered one commonality between the two activities studied: adults activated the right ventrolateral prefrontal cortex (VLPFC), particularly the rIFG, but children did not. In contrast to the modular hypothesis of inhibitory regulation, they discovered that the PFC areas (especially the rIFG) do not have a distinct inhibition area.

A subsequent study using the go/no-go paradigm found that successful behavioral inhibition was related with stronger activations in the prefrontal and parietal areas for children, compared with adults. To manipulate the difficulties of the task demands, Durston et al. (2002) varied the number of go trials that preceded no-go trials. This type of manipulation allowed for a comparison of children’s and adults’ performance on trials of similar difficulty. More importantly, researchers might change the prominence of the interfering information to see how much immature inhibitory control capacity is defined by vulnerability to interference. In adults with increased interference from go trials, successful inhibitory control was linked to increased activities in the VLPFC, right parietal lobe, and right DLPFC regions. When blocking a behavioral response in children, however, the networks showed the maximum level of activation, regardless of the number of previous responses (Durston et al., 2002). In addition, activations in the ventral-striatal areas were linked to age and performance. The findings show that children are more vulnerable to interference than adults, which may be due to variations in underlying frontal-striatal circuits.

Another recent study employed functional magnetic resonance imaging (fMRI) to ask children (years 10–12) and adults (ages 18–26) while they performing the marble challenge, in which they had to choose between acting on and blocking a prepotent reaction while fMRI data was recorded (Schel et al., 2014). The right hemisphere’s purposeful inhibition was linked to the activation of the fronto-basal ganglia network. The STN and the right dorsal fronto-medial cortex, which had previously been associated to intentional inhibition and purposeful behavior, had similar levels of activation. Despite the fact that both children and adults actively restrained their behavior to the same degree, children showed higher activation in the right fronto-basal ganglia network, but not in the STN or the dorsal fronto-medial cortex, during intentional inhibition.

Furthermore, there was a link between self-reported impulsivity and deliberate inhibition. The neurological underpinnings of early childhood response inhibition were explored by Rahman et al. (2017). Five-year-old youngsters did a go/no-go task with or without time pressure while scalp EEG was recorded (fast vs. slow condition). In comparison to go trials, when inhibition was required on no-go trials, the left frontal N2 and posterior P3 were elevated. Time pressure affected the early-occurring P1 variable, which was detrimental to behavioral performance. Similar to the N2, the topography of the heightened no-go P3 identified in their sample differed significantly from that documented in adult studies. In their early study on children, the heightened no-go P3 was detected at posterior midline electrode sites, whereas in adults, it is frequently shown at frontal midline electrode sites, a phenomenon known as “no-go anteriorization” (Fallgatter and Strik, 1999).

At posterior electrode sites, adults display a more significant go P3 than no-go P3, indicating that they are paying attention to targets (Bruin et al., 2001). Adults show a posterior P3 in the oddball task, indicating that they are processing infrequent targets (Friedman et al., 2001; Gaeta et al., 2003). Although it is possible that the P3 impact observed in this study is an outlier, we feel it is unlikely to be the result of rare target probability because individual go and no-go stimuli were delivered at the same frequency. The change in the topography of the no-go P3 identified in Rahman et al. (2017) study could represent children’s reliance on additional posterior brain regions in the right hemisphere to facilitate response inhibition. Similar results have been reported in middle childhood when it comes to N2 (Jonkman et al., 2007). Jonkman et al. (2007) discovered that in adults, right medial frontal sources were enough to explain the brain activity underpinning response inhibition, but that in children, additional right posterior sources were required. The neural networks governing inhibitory processes evolve from a more posterior, dispersed structure to a more frontal, concentrated pattern as children get older, according to studies utilizing brain imaging technologies (Bunge et al., 2002; Durston et al., 2006).

Lo et al. (2013) used EEG indices to study age-related changes in response inhibition in preschool children. They suggested that N2 amplitude and beta and gamma power are important indicators of inhibitory control ability. In preschoolers aged 5–6 they showed a positive correlation between electrophysiological components and behavioral improvement in inhibitory control. In particular, they observed a significant improvement in response inhibition in 6-year-olds over 5-year-olds. Moreover, they discovered an increase in the right frontal beta power during successful stop trails. This shows that age-related variations in response inhibition may reflect preschool children’s comparatively undeveloped frontal brain development.

Moreover, neurological and psychiatric diseases were analyzed in other studies with health controls (Tremblay et al., 2020). For example, Tremblay et al. (2020) elucidated the brain mechanisms of an important cognitive deficit in ADHD, identifying potential white matter tracts related to deficient inhibitory control. Twelve adolescents with ADHD and twelve age-matched healthy controls (age range 9–18) were scanned while performing the stop signal task (SST). Reactive inhibition activated the right inferior frontal gyrus (IFG) in both groups. ADHD participants recruited the IFG bilaterally. Prospective inhibition preactivated the same area of the right IFG that was activated during reactive inhibition in controls. In ADHD participants, prospective inhibition was associated with deactivation in this region. Controls also deactivated the right medial prefrontal cortex (rMPFC) during prospective inhibition, whereas ADHD participants activated the same area (Bhaijiwala et al., 2014; Tremblay et al., 2020).

Moreover, children with deficits in behavioral inhibition face increased risk for social anxiety (Thai et al., 2016). However, not all children with behavioral inhibition develop anxiety symptoms. Inhibitory control has been suggested as a moderator of the pathway between behavioral inhibition and social anxiety. In fact, Troller-Renfree et al. (2019) suggested that inhibitory control development in childhood occurs independent of behavioral inhibition levels. However, rapid increases in inhibitory control performance moderate risk for social anxiety symptoms in children with behavioral inhibition (BI) deficit. Notwithstanding, Triplett et al. (2014) found that children with epilepsy demonstrated impaired AS performance compared to controls during both neutral (no reward) and reward trials but exhibited significant task improvements during reward trials. *Post-hoc* analysis revealed that younger patients made more errors than older patients and all controls. fMRI results showed preserved activation in task-relevant regions such as bilateral frontal and supplementary eye fields, putamen, and precuneus in patient and controls, with the exception of increased activation in the left posterior cingulate gyrus in patients, specifically with generalized epilepsy across neutral and reward trials.

#### Teenagers

Inhibitory control ability is especially important in teenagers, as adolescence is a period of time that coincides with responsibility, peer interactions, and social awareness (Vara et al., 2014). Moreover, inhibitory control plays a central role in these evolving social executive functions in adolescents (Ellis et al., 2004; Crone et al., 2008; Pharo et al., 2011; Vetter et al., 2013). Poor inhibitory control development may have negative impacts on adolescent socialization processes (Vara et al., 2014). For example, Gligorović and Buha Ðurović (2014) suggested that inhibitory control represents a significant developmental factor of different adaptive behavior domains in children with mild intellectual disability.

There are a bulk of literature suggesting the developments in the frontal lobe maturation and white matter over the adolescence (e.g., Gogtay et al., 2004; Sowell et al., 2004; Paus, 2005; Shaw et al., 2006). Some of the earlier studies found the recruitment of the unilateral prefrontal cortex (Tamm et al., 2002, 2004), and others found a bilateral activation pattern of the prefrontal cortex (Durston et al., 2006; Smith et al., 2006; Carrion et al., 2008). Moreover, it seems that cross-network integration, predominantly of the cingulo-opercular/salience network, increased with age. Importantly, this augmented integration of the cingulo-opercular/salience network significantly moderated the robust effect of age on the latency to initiate a correct inhibitory control response (Marek et al., 2015). Furthermore, Tamm et al. (2002) used the go/no-go task on participants aged from 8 to 20 years old they detected a positive correlation between age and activities in the left IFG, insula, and orbitofrontal gyrus. However, there was a negative relationship between age and activations in the middle and superior frontal gyri. Similarly, in a later fMRI study using SST, Rubia et al. (2007) found a positive correlation between age and activities in bilateral inferior frontal cortex. Moreover, there were enhanced activities in the right inferior prefrontal cortex in adults compared to adolescents. Thus, right frontal regions were found to be reliably involved during inhibitory control. Velanova et al. (2008) scanned 77 people aged between 8 and 27 years old with fMRI while asking them to perform an oculomotor task that requires inhibitory control ability. They found that age-related increases in performance are attributed to functional changes in the dorsal anterior cingulate cortex (dACC) linked with error management and error-feedback use, as well as changes in the recruitment of attentional networks. In adults, dACC showed higher and prolonged modulation for mistake vs. correct trials, but drastically reduced in children. Activities in posterior attentional areas were low in younger age groups. This could be expected because of on higher level of activations of prefrontal cortical regions.

One longitudinal fMRI study using the AS found that the prefrontal engagement was at the max during childhood, but by adolescence, the right DLPFC engages at adult levels. The activities in the right dACC showed increased engagement throughout adolescence, which mediate behavioral developments in AS performance (Ordaz et al., 2013). Age-related development in anterior cingulate functions has also been used to describe SST using fMRI and EEG (Segalowitz et al., 2010; Ferdinand and Kray, 2014). The dACC was also found in performance monitoring tasks, and works as an altering system to engage cognitive control systems (Cavanagh and Frank, 2014). Thus, immaturities in error processing during adolescence may also impact the limitations in inhibitory control tasks. Another longitudinal neuroimaging study revealed that higher externalizing scores were associated with developmentally stable hypo-activation in the left middle frontal gyrus, but divergent developmental pattern of left posterior parietal cortex activation, suggesting that early adolescence may be a unique period of substance use vulnerability via cognitive and phenotypic disinhibition (Kim-Spoon et al., 2016; Quach et al., 2020). Studies about eating disorders in adolescents suggested that failed inhibitory control is an early marker of those disorders (Bartholdy et al., 2019; Cope et al., 2020). In particular, Bartholdy et al. (2019) found greater recruitment of the medial prefrontal and anterior cingulate regions during failed inhibition accords with abnormal evaluation of errors contributing to disordered eating behavior development.

Similarly, another fMRI study on 290 participants used a go/no-go task, with 88 patients undertaking repeated scanning at 1- to 2-year intervals (Cope et al., 2020). One group (N = 117) were scanned when they were 7–13 years old, while the other were scanned when they were 18–23 years old (N = 173). There were two patients with a substance use disorder (SUD) in 33.1% of the study, one patient with a SUD in 43.8%, and no patients with a SUD in 23.1%. There were 1,162 scans performed, ranging in age from 7 to 28, with longitudinal data from the cohorts overlapping between the ages of 16 and 21. To describe voxel-by-voxel shifts in hemodynamic response associated with efficient inhibitory control, Cope et al. (2020) used a marginal model with sandwich estimator standard errors. Cope et al. (2020) discovered that age was correlated with strong positive linear activation in the frontal, temporal, parietal, and occipital cortices in the left hemisphere. Negative linear, positive or negative quadratic, or positive or negative cubic comparisons resulted in no clusters survived thresholding.

A recent study found that inhibition and intrinsic functional brain architecture could be influenced in their relationship by age and emotional function (Petrican and Grady, 2019). Using fMRI dataset from Human Connectome Project (N = 359 participants) and Nathan Kline Institute-Rockland lifespan sample (N = 247), they found that between subjects with superior affective functioning, adolescence and old early adulthood with better inhibitory control correlated to brain pattern that typified processing of motivationally salient information, with a stronger resting state expression. On the contrary, after the age of 49, the reverse impact developed. Moreover, a substantial relationship between inhibition and brain architecture occurred just before the age of 28 in people with lower emotional functioning. Superior inhibition was related to the neural pattern of effortful cognitive processing in this group, also with higher resting state expression. Therefore, their findings suggest that motivational relevance contributes significantly to excellent cognitive functioning during the early stages of development.

Banich et al. (2019) investigated how cognitive control affects the processing of both task-relevant and task-irrelevant information. During adolescence, cognitive control areas in the prefrontal cortex can block sensory cortex, influencing processing of task-irrelevant information. The processing of task-relevant information could potentially impact performance, although no evidence of such processing was found to be controlled by cognitive control areas (Banich et al., 2019). The purpose of their study was to see if stimulation of the dorsolateral prefrontal cortex (DLPFC) changes in reaction time by modifying processing of task-relevant/task-irrelevant information in posterior brain areas (Banich et al., 2019). The amygdala, a brain area involved in the processing of salient task-irrelevant emotional input, was subjected to the same procedure. They discovered that greater DLPFC activity on a given trial was linked with decreased perceptual processing of the task-irrelevant face, supporting the hypothesis that top-down cognitive control might modify processing of task-irrelevant information (Banich et al., 2019).

Wang et al. (2021) used a large-scale longitudinal dataset, which consist of adolescents aged from 14 to 19, to track their inhibitory control development. During fMRI sessions, individuals in their research performed a SST. They attempted to identify brain traits that predict development by building prediction models inside a network or between two networks (Wang et al., 2021). In particular, they found that interconnections between ventral attention (VAN) and subcortical networks might predict individual inhibitory control development and construct a prediction model that extended to previously undiscovered people. Individual disparities in inhibitory control development are shown by their research. Additionally, within 5 years, they discovered that connection between these two networks was associated to drug misuse difficulties (Wang et al., 2021). Therefore, they hypothesized that early neural predictors of development might offer a neural foundation for early customized therapies to prevent inhibitory control impairments in adolescents. According to these results, functional connection of network analyzed (VAN and subcortical networks) could predict future drug misuse, revealing a feasible therapeutic translation pathway for this neurological predictor (Wang et al., 2021).

On the other hand, magnetoencephalography (MEG) offers a temporal resolution and the millisecond level, which allows characterization of electrophysiological activities generated by neuronal dynamics during different phases of cognitive processing. One MEG study on inhibitory control development focused on the differences in the preparatory period between adolescents and adults and found that there were significant differences in oscillatory activities in adolescents compared with adults, which also corresponds with performance (Everling and Munoz, 2000; Hwang et al., 2016). Specifically, adolescents demonstrated adult level beta-band power, but lower alpha-band power and beta in the frontal eye field (FEF). Moreover, there were beta/alpha DLPFC/FEF cross-frequency couplings. Previous research has found that beta rhythms are related to cortical glutamatergic functions in deep layers (Roopun et al., 2010) via top-down inputs activating pyramids neurons (Constantinidis and Luna, 2019). Moreover, Alpha rhythms are linked to dampening of neuronal activities through inhibitory processes (Jensen and Mazaheri, 2010), which support inhibition in the AS task (Belyusar et al., 2013).

A subsequent study used MEG to investigate the spatial temporal neural characteristics for behavioral inhibition during a go/no-go task in both teenagers and adults. Vidal et al. (2012) focused on the spatial temporal activities of neural responses during a go/no-go task in 14 teenaged and 14 adults. They controlled the task complexity of both groups. Results demonstrated bilateral prefrontal activities during inhibitory control in both groups. However, they had different temporal spatial patterns. There was an increased activity in the middle frontal gyri in teenagers at around 300 ms after stimulus onset, but at around 260 ms in the IFG in adults. Furthermore, the inhibition of a prepotent response demonstrated a stronger involvement of the left hemisphere in teenagers compared with adults.

A more recent study used MEG and co-registered magnetic resonance imaging (MRI) to investigate the neural basis underlying the inhibitory control abilities in teenagers with that of adults (Vara et al., 2014). Specifically, Vara et al. (2014) recruited 15 adolescents and 15 adults to do the go/no-go task. There were two conditions in the experiment: control condition (go: no-go trials = 2:1), and an experimental condition (go/no-go trials = 1:2). Vara et al. (2014) compared acquired images of brain activations between both the teenager group and the adult group. The results revealed the recruitment of the rIFG in adults, but delayed recruitment of the left inferior frontal gyrus (lIFG) in adolescents. In addition, adolescents recruited the right middle and superior temporal gyri, nonetheless adults activated the right temporal gyrus for a much brief duration. Together, these results suggest that adolescents are able to demonstrate adult-level inhibitory control behaviorally. However, the prefrontal systems in adolescents do not reach full maturity, so do not engage readily in inhibitory control, thus undermine optimal inhibitory control.

### Neurodevelopmental Diseases Related to Inhibitory Dysfunctions

Impaired executive functioning is thought to be the cause of impulsivity. Impulsive behavior is described by the presence of dysfunctional inhibitory mechanisms and strong “impulsions” (or impulses), as well as being triggered and modulated by dispositional and situational factors (Metcalfe and Mischel, 1999; Bari and Robbins, 2013). There would be no need for inhibition if there were no strong desire, impulse, or habit, but fully functioning inhibitory mechanisms would avoid the impulsive act. Neurodevelopmental disorders such as obsessive-compulsive disorder (OCD; Mancini et al., 2018); ADHD (van Hulst et al., 2018); autism (Schmitt et al., 2018); motor stereotypes (Mirabella et al., 2020) all include impulsive characteristics.

Mirabella et al. (2020) found that children with primary motor stereotypies (i.e., stereotypies not associated with other neurological conditions) have a significant deficit in reactive inhibition as compared to normally developing children. However, the proactive control performance was similar to the normally developing control group. This proof may clarify the two main features of the primary motor stereotypies phenotype. On the one hand, patients’ inability to refrain from performing stereotypic movements when caused by excitement, tension, boredom, or fatigue may be due to a lack of reactive inhibition (Gao and Singer, 2013). On the other hand, an intact proactive control ought to permit patients to know about the unique circumstance and therefore stop when their focus is diverted (Gao and Singer, 2013). Interestingly, the findings of Mirabella et al. (2020) are complementary to those of Schmitt et al. (2018), who studied a large cohort of patients with simple autism spectrum disorder (ASD; i.e., patients with ASD without comorbid mental disorders) and found a deficiency in proactive control strategies while reactive inhibition was comparable to that of normally developing controls (Padmanabhan et al., 2015; Schmitt et al., 2018; Voorhies et al., 2018). Excitingly, the magnitude of restrictive, repetitive behaviors and, in particular, motor stereotypies were used to scale disability in proactive control.

Schmitt et al. (2018) took a similar experimental approach comparing to Mirabella et al. (2020), in the variant of the SST used in both cases was minimally demanding in terms of attentional and working memory demands. Hence, apart from the fact that participants were required to suppress key-press movements in the first study, while in the second study, participants were required to suppress arm-reaching movements, the differences in results are unlikely to be explained by experimental design. The age range of participants in the Schmitt et al. (2018) sample was much broader than in the Mirabella et al. (2020) sample, represented by a narrower age range. Since inhibitory control varies over the lifespan (Dupuis et al., 2019), a broad age range is more likely to generate large variability. Schmitt et al. (2018) found that proactive control improves in people with ASD and typically developing people during infancy, adolescence, and early adulthood. However, the ability to use preventive methods is hampered from infancy onward, and it grows more slowly in people with ASD than in people who are normally developing. Given these considerations, it is more likely that ASD and primary motor stereotypies will exhibit a distinct pattern of inhibitory control impairment. Individuals with ASD may be unable to learn to use contextual signals to suppress unwanted repetitive behaviors due to a deficiency in proactive inhibition. Behavioral inflexibility is a phenotypic feature of ASD, particularly in a novel or unexpected circumstances (Condy et al., 2019). Impaired proactive control may also indicate an intolerance to uncertainty, to which patients with ASD respond with restricted, repetitive behaviors (South and Rodgers, 2017). All of these exciting hypotheses will need to be investigated further.

Another important piece of evidence about the complexities of the consequences of inhibitory control deficits comes from van Hulst et al. (2018), who found a selective deficiency in reactive inhibition, but intact proactive control in children with ASD and comorbid ADHD and ADHD only when compared to normally developing children. The difference between these and the findings of Schmitt et al. (2018) can be explained by the fact that van Hulst et al. (2018) included patients with ASD who had severe ADHD symptoms. As a result, ADHD symptomatology was likely to be common, at least in terms of inhibitory control deficits.

Selective impairment of reactive inhibition seems to be a hallmark of ADHD, as found by Pani et al. (2013) using a standard key-press version of the stop-signal task. This feature is common to both inattention and hyperactivity ADHD subtypes (Pani et al., 2013; van Hulst et al., 2018). Furthermore, ADHD patients did not appear to have low-level automatic motor inhibition deficits. In theory, a reactive inhibitory deficiency may explain a variety of ADHD phenotype characteristics, including an inability to stick to tasks and continuously changing activity, failure to wait for one’s turn, and impulsive behavior. These behavioral traits should not be based on stimulus affordances (Keute et al., 2018), but rather on environmental or internal clues that children with ADHD are unable to avoid, even though they should be aware of the inappropriateness of their behaviors if proactive control is intact. Although the parallel deficiency in other executive functions, primarily attention makes the ADHD phenotype much more complex and disabling, this pattern of inhibitory control deficits which mimic that of primary motor stereotypies.

Mennes et al. (2012) tested 17 normally developing controls (TDC) and 17 age-matched children with ADHD, aged from 8 to 13 years, using resting-state fMRI. In their study, two related inhibition indices were examined: stop signal reaction time (SSRT) and stop signal delay (SSD). The first one measures inhibitory speed, whereas the second measures inhibitory success (Mennes et al., 2012). Independent of diagnosis, SSRT and SSD demonstrated connectivity–behavior correlations. They discovered that children with ADHD had distinct connectivity–behavior associations than children with TDC. Their findings showed that resting state functional connectivity techniques may be used to analyze brain/behavior interactions and reveal pathology-related changes in neural circuit contributions to cognition and behavior.

Tourette syndrome and OCD are the other two disorders that are characterized by impaired inhibitory control. The cognitive processes underlying tic and compulsion regulation have been shown to be entirely different. Mancini et al. (2018) used a reaching arm variant of the SST to determine reactive and proactive inhibitory regulation in a large cohort of drug-naive children and adolescents with Tourette syndrome, OCD, or both. Both reactive and proactive inhibition were found to be impaired, and the severity of the disorder scaled with the severity of OCD symptoms. However, in patients with uncomplicated Tourette syndrome, inhibitory control was similar to that of normally developing controls. Patients with OCD are unable to avoid performing compulsive acts caused by their intrusive thoughts due to a combined imbalance in proactive and reactive inhibition, greatly limiting their ability to learn how to suppress impulses in the same or similar circumstances. Patients with OCD may be aware of a less efficient cognitive regulation of motor responses in the short and long term, which may play a role in the development of anxiety, depression, and maladaptive beliefs like danger overestimation, intolerance of ambiguity, and fear of losing control of their behavior (Mirabella, 2021). The extreme damage to inhibitory control systems may also explain why OCD symptoms last much longer than tic symptoms in adulthood (Bloch et al., 2006).

A tic is characterized by a rapid, repeated, non-rhythmic muscular action or vocalization involving specific muscle units (American Psychiatric Association, 2000; Leckman et al., 2006). Tic disorders are classified according to the type (motor or phonic) and duration of tics in the Diagnostic and Statistical Manual of Mental Disorders (DSM; American Psychiatric Association, 2000). Recently, Cothros et al. (2021) investigated inhibitory control in children with tics by presenting many mobile stimuli at the same time. They analyzed 64 children (aged from 7.5 to 18.5 years old) with tics compared to 146 developing controls (aged from 6.1 to 19.9 years old), during object-hit-and-avoid task using Kinarm (robotic bimanual exoskeleton protocol; Cothros et al., 2021). In particular, participants sought to strike just the targets while avoiding the distractor items as they traveled around a screen. They showed that children with tics (without concomitant ADHD) have a diminished capacity to inhibit reactions to possible triggers for action. This might be due to excessive sensorimotor noise or improper sensory gating (Cothros et al., 2021). However, the main finding of their study was that children with tic disorders hit more distractors than controls (Cothros et al., 2021). Jurgiel et al. (2021) examined brain oscillatory activity and effective connectivity in children (aged from 8 to 12 years old) with and without persistent tic disorder while performing a cognitive inhibition task using a case–control approach. They collected EEG data from their sample during the flanker task, finding that during incongruent flanker trials, children with chronic tic disorder showed substantial cerebral spectral power disparities (Jurgiel et al., 2021). In particular, there was less wide band oscillatory power modulation in the anterior cingulate cortex compared to controls. Additionally, they found that in comparison to controls, children with persistent tic disorder had increased involvement of the anterior cingulate and other fronto-parietal network hubs (Jurgiel et al., 2021). Specifically, they reported for the first time in chronic tic disorders (CTD) cortical source-resolved, event-related brain oscillatory dynamics and effective connection during inhibitory processing (Jurgiel et al., 2021).

As these disorders have very different neural underpinnings, the deficits in inhibitory control are more consistent with the network than with the modular view of inhibitory control (Snyder et al., 2015). In other words, this evidence is consistent with the network view of inhibitory control, thus there is not a common cognitive mechanism causing inhibitory deficits through different disorders.

## Concluding Remarks

To conclude, we systematically reviewed brain changes that relate to inhibitory control performance in infants, toddlers, children, and teenagers. We presented evidence against a modular account of inhibitory control and propose that inhibitory control is a broad class of cognition, which is supported by a large-scale frontal-parietal network. Specifically, we proposed that the development of inhibitory control is related to the recruitment of core inhibitory control networks rather than a single region like the rIFG. We additionally surveyed the neurodevelopment dysfunctions associated with inhibitory control across development, which was previously only noted in healthy adults.

Our proposed shift to understand inhibitory control as a result of the communication of a large-scale network especially during development, which provides insights for future basic as well as clinical research. For example, subsequent research could investigate whether the performance in inhibitory control tasks could be a biomarker for various psychiatric and neurological conditions during development. In addition, future studies may also focus on two specific directions: (1) separate three processes in inhibitory control including interference resolution, action withholding, and action cancelation during development. (2) As functional and structural changes are different in inhibitory control during aging (Hu et al., 2018), future studies should investigate structural and functional changes by using both structural and functional MRI during development and in different neurodevelopmental diseases characterized by poor inhibitory control.

## Author Contributions

WK: conceptualization, writing—original draft, writing—review and editing, and funding acquisition. SH: writing—original draft. MR, KV, and AM: writing—review and editing. All authors contributed to the article and approved the submitted version.

## Footnotes

1
https://pubmed.ncbi.nlm.nih.gov

2
http://www.prisma-statement.org
